# Supplementary figures and images for: Assessing migration patterns in Passerina ciris using the world’s bird collections as an aggregated resource
Source: PeerJ. 2016 Apr 7;4:e1871. doi: 10.7717/peerj.1871 (PMC4830235; doi:10.7717/peerj.1871)

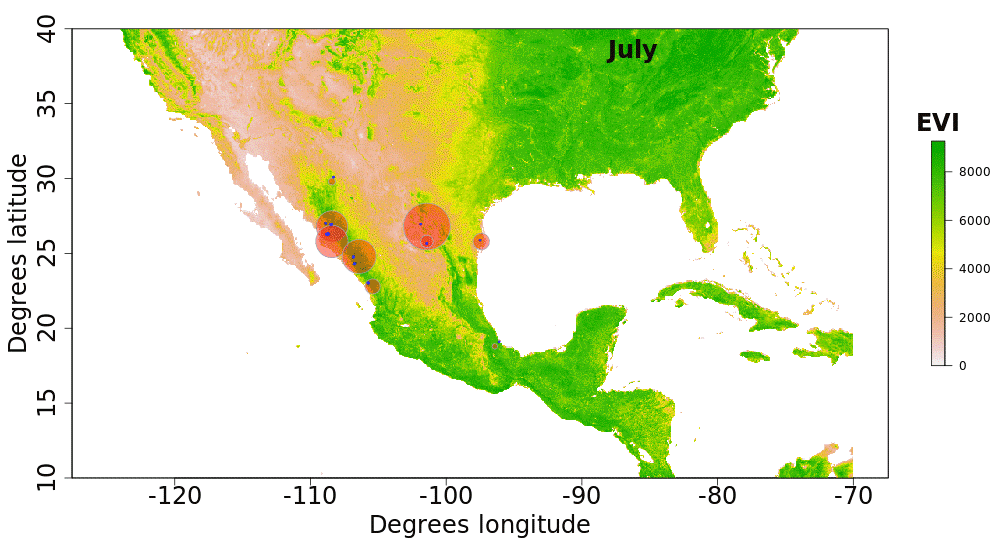

Supplement: Supplemental Information 1 — Figure 2 (.gif animation). Abundance index (AI) values for Passerina ciris specimens in Mexico by month, plotted against EVI analysis of remote sensing data. Red circles indicate the occurrence of P. ciris specimens, with the diameter of the circle proportional to value of Abundance Index. Green areas indicate high EVI values, correlated with regions with a high density of live green plants (photosynthetically active vegetation). [file peerj-04-1871-s001.gif]
